# A Typha Angustifolia-Like MoS_2_/Carbon Nanofiber Composite for High Performance Li-S Batteries

**DOI:** 10.3389/fchem.2020.00149

**Published:** 2020-03-03

**Authors:** Xingxing Gu, Han Kang, Chengbin Shao, Xiaolei Ren, Xiaoteng Liu

**Affiliations:** ^1^Chongqing Key Laboratory of Catalysis and New Environmental Materials, College of Environment and Resources, Chongqing Technology and Business University, Chongqing, China; ^2^Department of Mechanical and Construction Engineering, Faculty of Engineering and Environment, Northumbria University, Newcastle upon Tyne, United Kingdom

**Keywords:** Li-S batteries, MoS_2_/BCF, electrocatalysis, chemically trapping, polysulfides

## Abstract

A Typha Angustifolia-like MoS_2_/carbon nanofiber composite as both a chemically trapping agent and redox conversion catalyst for lithium polysulfides has been successfully synthesized via a simple hydrothermal method. Cycling performance and coulombic efficiency have been improved significantly by applying the Typha Angustifolia-like MoS_2_/carbon nanofiber as the interlayer of a pure sulfur cathode, resulting in a capacity degradation of only 0.09% per cycle and a coulombic efficiency which can reach as high as 99%.

## Introduction

Lithium-sulfur (Li-S) batteries attract considerable interest due to their high energy density (2,600 Wh/kg). As well as the cathode material, sulfur is cost-effective, naturally abundant, and environmentally friendly (Gu and Lai, 2019). However, Li-S batteries are plagued with various challenges. Among these, serious lithium polysulfide (LiPSs) shuttling—inducing large capacity degradation, severe polarization, sluggish reaction kinetics, and inefficient self-discharge—is one of the most significant issues (Liu et al., 2019; Xu et al., 2019).

In view of such a serious situation, tremendous efforts have been made to suppress polysulfide shuttling with physical confinement and chemical absorption by constructing various kinds of nanostructures, such as the non-polar porous carbon (Rehman et al., 2016; Guo et al., 2018), graphene (Yin et al., 2016), carbon nanotubes (Yang et al., 2018), as well as the polar metal oxides (Gu et al., 2016; Song et al., 2018), metal sulfides (He et al., 2019; Lin et al., 2019), metal carbide (Chen et al., 2018; Dong et al., 2018; Song et al., 2019), metal nitride (Jiao et al., 2019; Wang et al., 2019), etc. Accordingly, LiPSs shuttling has alleviated to some extent. Recently, researchers focused on the electrocatalysis of reducing sulfur to LiPSs and oxidizing Li_2_S_2_/Li_2_S to LiPSs or even to sulfur during the charge-discharge process, which is important for achieving high reversible capacity and coulombic efficiency. By applying the electrocatalysis concept of enhancing the redox reactions of polysulfides, increasing numbers of catalysts suitable for redox conversion of lithium polysulfides have been reported (Jeong et al., 2017; Liu et al., 2018; Hao et al., 2019; He et al., 2019; Jiao et al., 2019; Lin et al., 2019; Yuan et al., 2019).

In this work, we synthesized a new 1D nanostructure: a Typha Angustifolia-like MoS_2_/carbon nanofiber composite as both a chemical trapping agent and redox conversion catalyst for LiPSs, to enhance the sulfur cathode performances. The sulfur cathode with the MoS_2_/carbon nanofiber interlayer illustrates an initial capacity as high as 926.1 mAh/g at a charge-discharge current of 0.5 C. Even after 300 cycles a reversible capacity of 661.5 mAh/g could maintain.

## Experimental

### Materials Preparation

Bamboo carbon fiber (BCF) preparation: the bamboo stick was immersed in 8 M KOH solution and hydrothermal reaction for 12h. Then the resultant bamboo fiber was dried and annealed at 800°C for 2h under Ar atmosphere. Finally, the BCF was obtained by washing with distilled water and drying overnight.

BCF/MoS_2_ preparation: 114 mg Ammonium molybdate tetrahydrate [(NH_4_)_6_Mo_7_O_24_•4H_2_O] and stoichiometric overdose thiourea were dissolved in 60 mL distilled water, then 40 mg BCF dispersed in the mixture solution by ultrosonication. Next, the solution was transferred into the Teflon autoclave and reacted for 12h at 200°C. At that time, a black composite was obtained. After washing with distilled water and ethanol and then drying, the composite was annealed in H_2_/N_2_ (5% volume percent of H_2_) atmosphere at 800°C for 1h to finally obtain the Typha Angustifolia-like BCF/MoS_2_ composites.

### Materials Characterizations

The samples' structures were characterized by X-ray diffraction (XRD) (Model LabX-6000, Shimadzu, Japan) and the JSM-7001F scanning electron microscope (SEM) (JEOL, Japan).

### Electrochemical Measurements

Sulfur, carbon black and polyvinylidene fluoride (analytical reagent, Sigma-Aldrich), in a weight ratio of 80:10:10, were mixed with solvent of 1-methyl-2-pyrrolidinone (analytical reagent, Sigma-Aldrich). After stirring for 12 h, the electrode slurry was obtained. Then the slurry was pasted on the Aluminum foil via the blade-coating method. After drying at 60°C in a vacuum oven overnight, the electrode was cut into wafers with a size of 0.5 cm^2^ and a weight of ~1.5 mg. The interlayer was made by BCF/MoS_2_, carbon black, and polytetrafluoroethylene in a weight ratio of 80:10:10 with solvent of 1-methyl-2-pyrrolidinone to form a flexible film. After drying at 60°C in a vacuum oven overnight, the film was cut into wafers with a diameter of 11 mm, thickness of 150 μm, and a weight of approximately 1.2 mg.

Then batteries were assembled in a glove box (Vigor, China), using lithium metal as the counter electrode, polypropylene (Celgard 2300) as the separator, and 1 M lithium bis (trifluoromethane)sulfonimide (LiTFSI) in 1,3-dioxolane/1,2-dimethoxyethane (DOL/DME) (1:1, v/v) containing 0.2 M LiNO_3_ as the electrolyte. And the BCF/MoS_2_ wafer could be placed between the separator and the electrode as the interlayer during the battery assembling process. Finally, the charge and discharge performances of the coin cells were tested with a LAND CT-2001A instrument (Wuhan, China) and the cyclic voltammetry (CV) curves were performed on a CHI 660D electrochemical workstation (CHI Instrument, Shanghai, China); in both the potential range was controlled between 1.5 and 3.0 V at room temperature. The capacities were calculated based on the sulfur mass. Additionally, the electrode impedance spectrums (EIS) were tested on CHI 660E (frequency range from 100 kHz and 10 mHz).

## Results and Discussions

**Figure 1:**
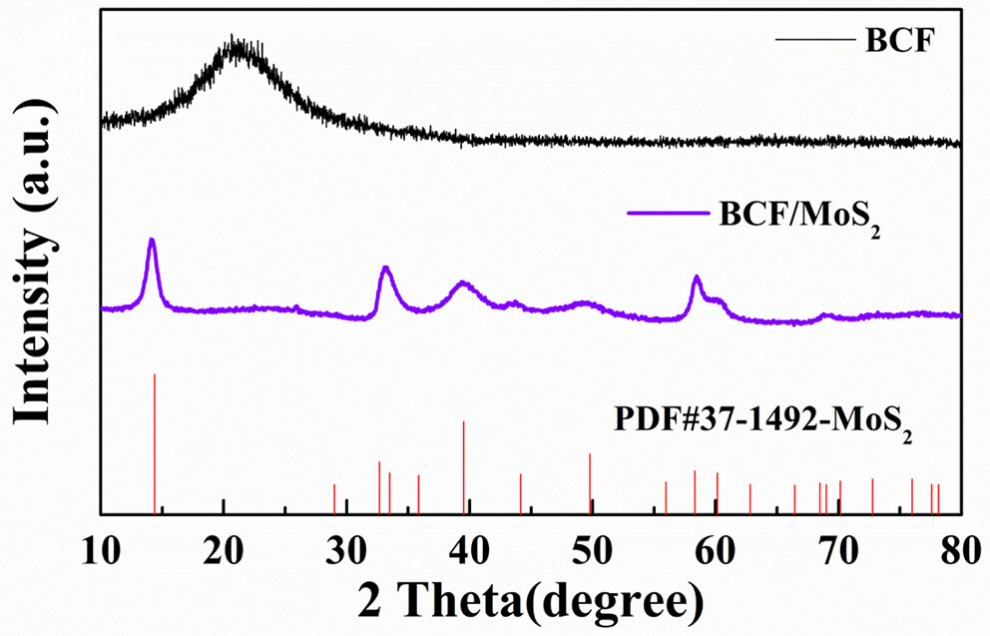
XRD spectra of BCF, BCF/MoS_2_, and Stand XRD spectrum of MoS_2_.

Firstly, the XRD was used to examine the crystallization structure of the synthesized product. As shown in Figure 1, The BCF/MoS_2_ has been successfully synthesized using a simple hydrothermal method. On the XRD spectrum of BCF, there is a wide peak at around 2 theta of 23°, which belongs to the partial graphitization of carbon, implying the good conductivity of BCF (Gu et al., 2015). While on the spectrum of BCF/MoS_2_, the peak belonging to the graphitization carbon has been covered by other strong peaks. All these peaks could be ascribed to the MoS_2_, and the crystal phase could match well with the MoS_2_ stand PDF card (37-1492).

**Figure 2:**
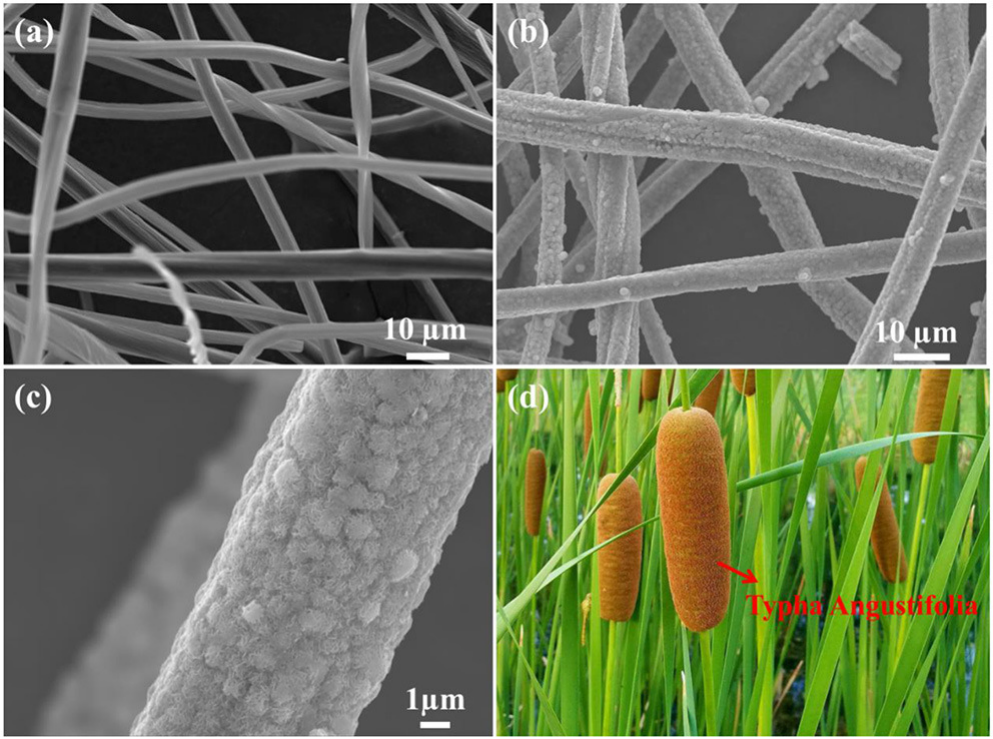
SEM images of **(a)** bamboo carbon fiber, **(b,c)** BCF/MoS_2_, and **(d)** the digital photo of Typha Angustifolia.

Following the morphology information of BCF and BCF/MoS_2_, they have been investigated by SEM. As shown in Figure 2a, the bamboo carbon with unique fiber structure has successfully synthesized. While in Figures 2b,c, the BCF as a core, and the MoS_2_ grown in the direction of the nanofiber line as a shell, has been observed. Such a unique one-dimensional structure is very much like Typha Angustifolia as shown in Figure 2d.

**Figure 3:**
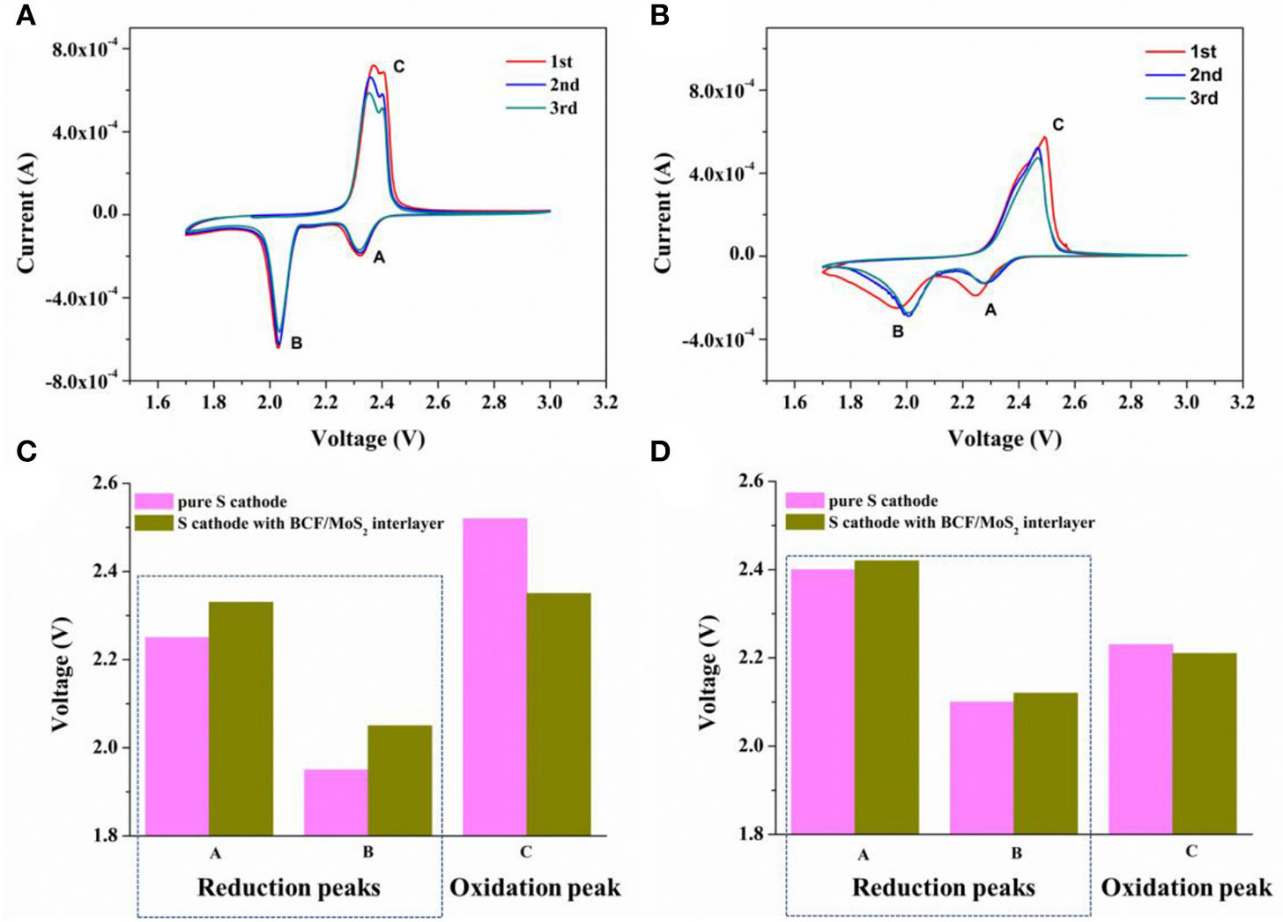
Kinetics of electrochemical reactions in Li-S batteries. CV test of **(A)** sulfur cathode with BCF/MoS_2_ interlayer and **(B)** pure sulfur electrode. Corresponding **(C)** peak potentials and **(D)** onset potentials of the sulfur cathodes with and without BCF/MoS_2_ interlayer from the first CV cycle in **(A,B)**.

Then the electrochemical performances of the sulfur cathode with and without the BCF/MoS_2_ interlayer have been investigated. As shown in Figure 3A, there are two obvious and stable redox peaks for the sulfur cathode with the BCF/MoS_2_ interlayer. While in Figure 3B, pure sulfur electrode (BCF/MoS_2_ interlayer) illustrates deformed and widened redox peaks in the CV curves, suggesting a sluggish kinetic process (Li et al., 2017; Liu et al., 2018). Comparing the peak potentials (Figure 3C) during the redox reactions, it is evident that the sulfur cathode with the BCF/MoS_2_ interlayer shows higher reduction potential and lower oxidation potential than that without the BCF/MoS_2_ interlayer, indicating that the BCF/MoS_2_ interlayer significantly lowers electrode polarization (Gu et al., 2015; Wang et al., 2018; He et al., 2019). This can be attributed to the catalysis effect of MoS_2_ on the oxidation/reduction of lithium polysulfides/Li_2_S (Wang et al., 2018; He et al., 2019). In terms of the onset potentials shown in Figure 3D, the onset potential of the sulfur cathode with the BCF/MoS_2_ interlayer in the oxidation reaction is ≈2.23 V, compared with ≈2.21 V for the pure sulfur cathode without the BCF/MoS_2_ interlayer. With respect to the reduction reaction, the onset potentials for sulfur cathode with the BCF/MoS_2_ interlayer are ≈2.42 and ≈2.12 V, compared with ≈2.4 and ≈2.1 V for the pure sulfur cathode without the BCF/MoS_2_ interlayer, which are lower by ≈20 mV. These results demonstrate that by inserting a conductive BCF/MoS_2_ interlayer, the redox kinetics are accelerated and the polarization losses significantly reduced for the Li-S battery (Gu et al., 2015; Li et al., 2017; He et al., 2019).

**Figure 4:**
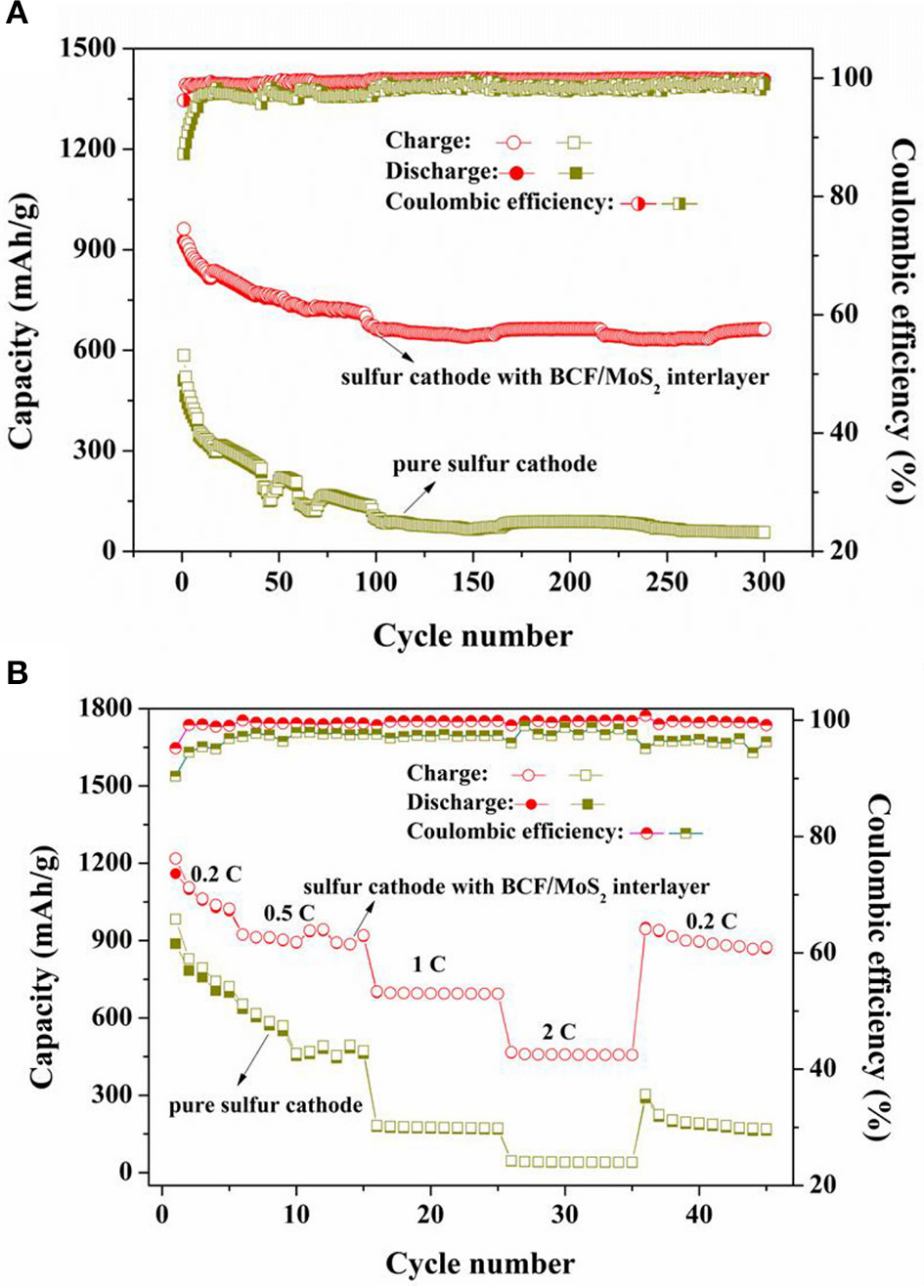
**(A)** The long-cycling performances of sulfur cathode with and without BCF/MoS_2_ interlayer at charge-discharge current of 0.5 C, **(B)** the rate capabilities of sulfur cathode with and without BCF/MoS_2_ interlayer.

Finally, we carried out the long cycling performances and rate capabilities of the sulfur cathode with and without the BCF/MoS_2_ interlayer. As shown in Figure 4A, the sulfur cathode with the interlayer shows a high initial specific capacity of 926.1 mAh/g. After cycling 300 cycles, it can still maintain a high reversible capacity of 661.5 mAh/g, and the capacity degradation rate is only 0.09% per cycle. However, the pure sulfur cathode without the BCF/MoS_2_ interlayer only demonstrates an initial capacity of 510 mAh/g and an extremely low reversible capacity of 56.3 mAh/g after 300 cycles. By contrast, the initial average discharge capacity of the pure sulfur cathode without the interlayer is ≈400 mAh/g lower than the sulfur cathode with the BCF/MoS_2_ interlayer, indicating significant dissolution and loss of LiPSs into the electrolyte during the initial cycles. Such severe dissolution and loss continues throughout the whole charge and discharge process because the ultimate reversible capacity is also extremely low. Additionally, from Figure 4B, it is clearly observed that the sulfur cathode with the BCF/MoS_2_ interlayer shows far better rate capabilities compared to the one without the BCF/MoS_2_ interlayer. Even if the charge-discharge current increases to 2 C a reversible capacity of around 456 mAh/g could still be reserved, and after the current switch to a low density of 0.2 C a recoverable capacity of approximately 900 mAh/g could be reached. Therefore, the BCF/MoS_2_ is highly effective as a polysulfide immobilizer for enhancing cycling life and rate capabilities (Gu et al., 2015).

What's more, it can be observed that the sulfur cathode with the BCF/MoS_2_ interlayer demonstrates an excellent coulombic efficiency (~99%), but the sulfur cathode without the interlayer shows an obvious weaker coulombic efficiency, particularly in the tens of cycles ahead. The coulombic efficiency results indicate that the BCF/MoS_2_ as electrocatalyst could significantly accelerate the redox reaction in Li-S batteries and improve coulombic efficiency (Gu et al., 2015; Jeong et al., 2017; Wang et al., 2018).

## Conclusions

In summary, the Typha Angustifolia-like MoS_2_/carbon nanofiber composite has been successfully employed as the interlayer in Li–S batteries. The BCF/MoS_2_ interlayer bestows Li–S batteries with excellent long-term cycle stability (only 0.09% capacity fade per cycle) and high coulombic efficiency (99%) even when the sulfur content is as high as 65% in the electrode. The exceptional performance can be attributed to: (1) the resultant conductive fiber networks, providing conductive skeletons for the electrons transfer; (2) abundant gaps and pores to store the sulfur; (3) polar MoS_2_ shell chemically trapping the LiPSs as well as catalyzing the LiPSs redox reaction. Therefore, the unique Typha Angustifolia-like MoS_2_/carbon nanofiber interlayer has shed a light on the development of high-performance Li-S batteries.

## Data Availability Statement

All datasets generated for this study are included in the article/supplementary material.

## Author Contributions

XG and XL designed this experiment. HK and XR conducted the experiment. XG and XL wrote the manuscript. All the authors participated in the discussions on the experiment results.

### Conflict of Interest

The authors declare that the research was conducted in the absence of any commercial or financial relationships that could be construed as a potential conflict of interest.
